# AgDataBox-IoT - application development for agrometeorological stations in smart

**DOI:** 10.1016/j.mex.2023.102419

**Published:** 2023-10-14

**Authors:** Antonio Marcos Massao Hachisuca, Eduardo Godoy de Souza, Wendel Kaian Mendonça Oliveira, Claudio Leones Bazzi, Diandra Ganascini Donato, Isaque de Souza Mendes, Mahuan Capeletto Abdala, Erivelto Mercante

**Affiliations:** a Technological and Exact Sciences Center, Western Paraná State University, Cascavel, Paraná, Brazil; b Computer Science Department, Federal University of Technology – Paraná, Medianeira, Paraná, Brazil

**Keywords:** Big data, Precision agriculture, Wireless communication networks, Internet of things, AgDataBox, AgDataBox-IoT-Meteology

## Abstract

Currently, Brazil is one of the world's largest grain producers and exporters. Agriculture has already entered its 4.0 version (2017), also known as digital agriculture, when the industry has entered the 4.0 era (2011). This new paradigm uses Internet of Things (IoT) techniques, sensors installed in the field, network of interconnected sensors in the plot, drones for crop monitoring, multispectral cameras, storage and processing of data in Cloud Computing, and Big Data techniques to process the large volumes of generated data. One of the practical options for implementing precision agriculture is the segmentation of the plot into management zones, aiming at maximizing profits according to the productive potential of each zone, being economically viable even for small producers. Considering that climate factors directly influence yield, this study describes the development of a sensor network for climate monitoring of management zones (microclimates), allowing the identification of climate factors that influence yield at each of its stages.

• Application of the internet of things to assist in decision making in the agricultural production system.
• AgDataBox (ADB-IoT) web platform has an Application Programming Interface (API).
• An agrometeorological station capable of monitoring all meteorological parameters was developed (Kate 3.0).

## Graphical abstract

**Figure:**
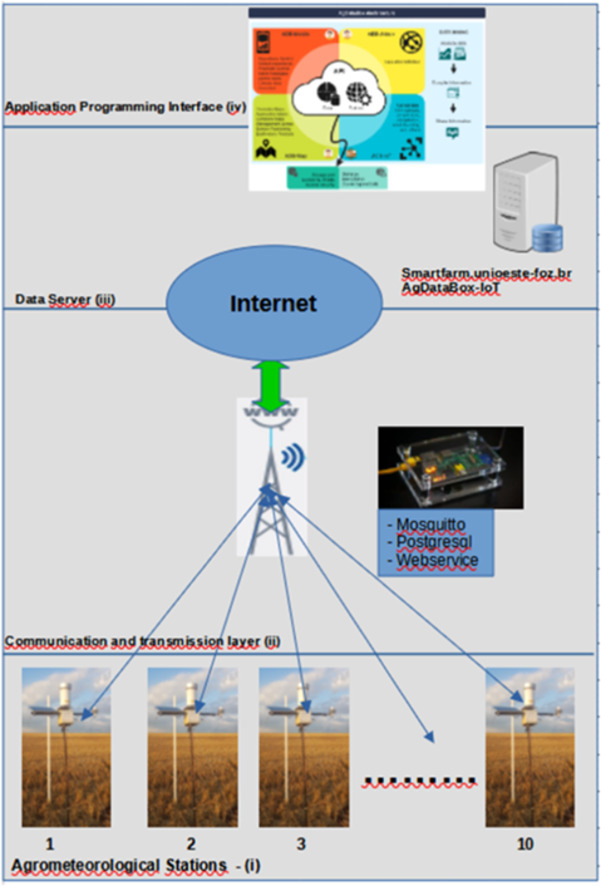

Specifications table

**Table:** 

Subject area:	Agricultural and Biological Sciences
More specific subject area:	Biometeorology, IoT, smart agriculture development
Name of your method:	AgDataBox-IoT-Meteology
Name and reference of original method:	N.A
Resource availability:	2.4 GHz WIFI technology, it was possible to reach a radius of 600 m between the station and the communication tower, but it is expected that with LoraWan, this range could be 10 km or more

## Background

Brazil is one of the main grain producers in the world, being the top country by soybean production and third-largest corn producer in the 2019/2020 growing season, only behind China and the USA. In 2020, Brazil had an area of soybean and corn cultivation of approximately 36.9 and 18.5 million hectares, respectively, equivalent to 124.84 and 102.5 million tons of grains [1]. As a result, agriculture has been consolidated as the main economic pillar of the State of Paraná in the last decade.

Soybean and corn are important protein sources in animal feed production, being indispensable for the chicken and swine production chains. The soybean planted area in the State of Paraná reached approximately 5.56 million hectares in the 2020/2021 growing season, with an estimated production of 20.5 million tons. Also, the State of Paraná reached record production in the 2019/2020 growing season, with a value of 20.8 million tons, representing about 17% of the Brazilian production [2]. Corn production in the 2019/2020 growing season reached about 15.2 million tons, and the cultivated area increased by 17% from 2010 to 2020, which is due to the second crop, as the first crop has been undergoing constant reductions [3].

Food production is expected to increase by around 70% by 2050 to meet the demand for food needs due to population growth, considering the extensive use of natural resources in agriculture, such as water, for future sustainability [4]. Therefore, there is a need for the successful development of efficient management practices and coherent use of resources, with the prediction of possible climate impacts being imperative, making real-time monitoring of climate and soil necessary [5].

Maintaining consolidated family farming in the countryside is essential for agri-food development. Technology assists in this process, but, on the other hand, there is the obstacle of the high cost of new technologies. The challenge is to make these tools accessible to medium and small farmers, even considering that connectivity in the field is the main obstacle in remote areas. There is also a high investment in equipment and sensors.

[6] observed that climate variables have a significant economic effect on wheat, corn, and rice yield. The authors found that moisture is beneficial to the three crops, with a 1% increase in the mean moisture during the growth stage increasing rice, wheat, and corn yield by 0.75, 0.96, and 0.61%, respectively.

Thus, the concept of Smart Farm arises. It is a system composed of sensors that monitor climate, soil, plant, and machine variables, powered by solar energy and transmitting data in real-time to the Internet, and is considered a smart farm technology for farmers [7].

The use of smart farms is an innovative approach that aims to increase yield and quality of agricultural products, increase the quality of life of farmers, reduce the need for labor, pesticides, and fertilizers, and making possible to check the environment of crop and livestock life promptly, anytime and anywhere, based on accurate data on crop growth and environmental information [8].

Smart farms are characterized by a device network (sensors) connected to the internet. This concept is called the Internet of Things (IoT) and refers to the interconnection of physical objects (e.g., vehicles, buildings, embedded technology, and sensors) with the internet, allowing for device management. IoT technologies enable various devices for data collection, transmission, and storage, which can be analyzed and used in decision-making when combined with algorithms to support user control activities [4,8].

Climate change has been a threat to the agricultural sector in many countries due to its irreversible impacts on global agri-food production systems and ecosystems [9]. Therefore, the constant monitoring of temperature, air humidity, solar radiation, wind speed, and soil moisture in the cultivated fields is necessary. One of the ways to improve these concepts is to encourage the use of technologies associated with precision agriculture (PA).

**Fig. 1:**
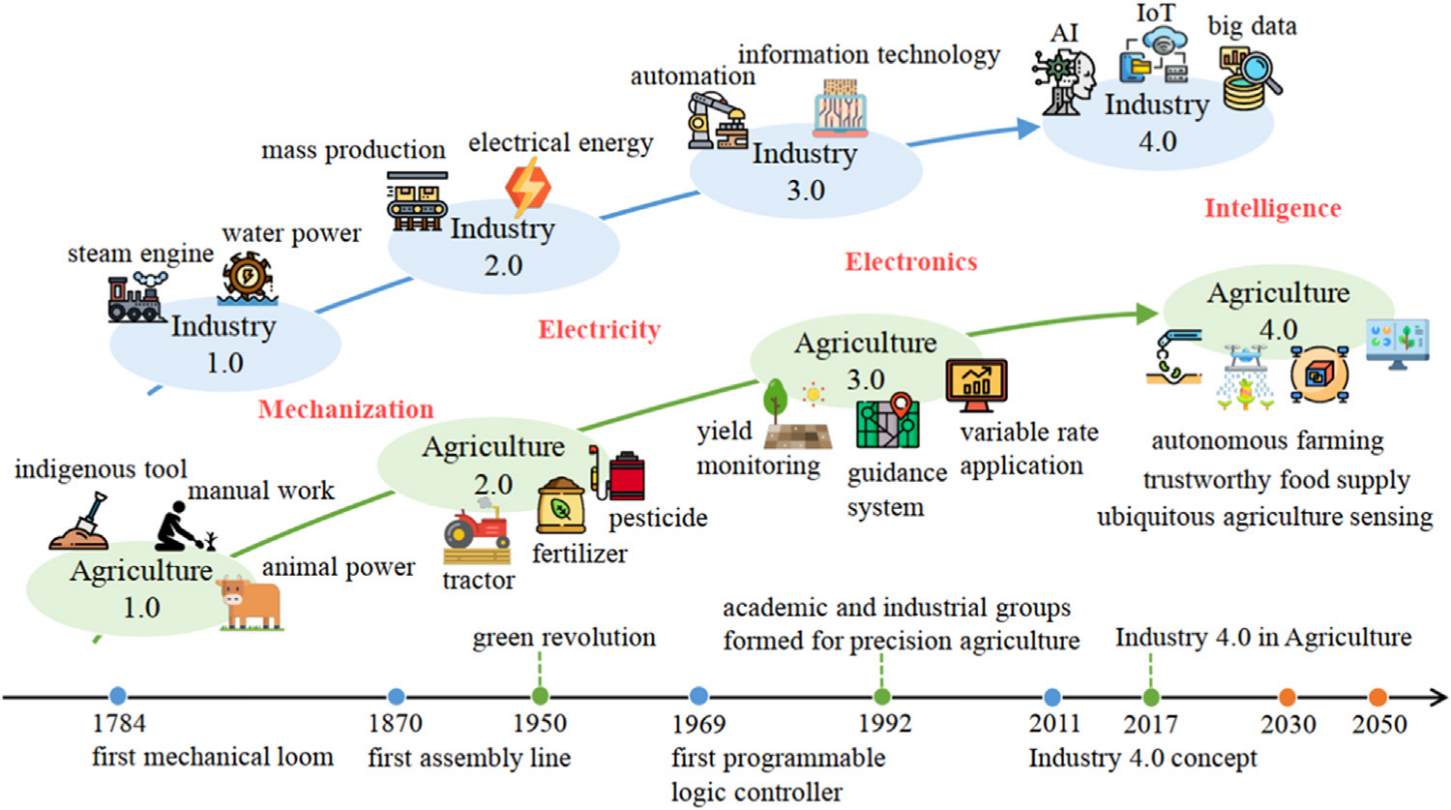
Development of agricultural and industrial revolutions. Source: [10].

The three previous industrial revolutions profoundly transformed the agricultural industry from native to mechanized agriculture and the recent PA (Fig. 1). The industrial agriculture paradigm significantly improves yield, but a series of challenges have gradually emerged, which have worsened in recent years. Industry 4.0 is expected to reshape the agricultural sector and promote the fourth agricultural revolution, agriculture 4.0, or digital agriculture (DA) [10]. Among many of its branches, agriculture 4.0 uses sensors and equipment capable of monitoring in real-time climate and water conditions at all stages of a plant in a management zone (microclimate), from planting to harvesting, calculating the degree-days, evapotranspiration, and generating data and information that allow the producer to manage the crop in a localized way and avoid water deficit and pesticide application if possible.

The availability of a wireless sensor network installed in the smart farm has led to obtaining a wide range of data (Big Data), resulting in the need for investments in a data management system (appropriate platform), storage, processing, visualization of information, and interaction of the data with the user [11]. After analysis, the collected information enables decision-making in real-time, besides predicting future needs, such as the need for water and irrigation systems [12]. Another aspect of climate monitoring is predicting the occurrence of pests and diseases and the development of management strategies [13].

The possibility of developing approaches that assist in decision making by the producer with the use of early warning systems or decision support systems has driven the development of monitoring systems through stations that enable the provision of early information about risks to crops related to seasonal factors, such as droughts and dry spells or even pests and disease vectors [14]. Temperature, precipitation, and humidity, among other climate factors, influence the occurrence of diseases and pests in crops, thus being crucial to an increase in the economic performance of this environment [15]. For instance, wheat is affected by various diseases worldwide, with pathogens being directly affected by climate conditions. Drought and high temperatures aggravate the interactions between wheat and its pathogens [13].

Global climate change is the current main challenge in intelligent monitoring of agrometeorological variables, as they have been changing the behavior of temperature distributions and precipitation distributions and variability during the seasons, making climate observation data insufficient for meteorological forecasts, as the climate has been behaving in a non-stationary manner, showing the need for monitoring and identifying local climate behavior patterns [16].

DA uses PA technology, smart networks, and data management tools. The objective of DA is to use all available information and knowledge to enable the automation of sustainable processes in agriculture. DA has achieved considerable development over the past two decades due to the availability of cheaper and more powerful sensors, actuators and microprocessors, high bandwidth cellular communication, cloud communication, and Big Data. Therefore, the flow of information comes from the used agricultural equipment and new services being offered with new algorithms, which transform data into valuable intelligence [17].

In this new DA paradigm, large amounts of data are made available, and the challenge is to add value to them with the insertion of data portals and work platforms. In data portals, end consumers can view their data without having to enter them manually. On the other hand, platforms allow consumers to transform data into new and more robust information. Thus, the passage from Agriculture 3.0, characterized by the use of PA, to Agriculture 4.0, as an evolution from PA to DA, requires the availability of specific portals and platforms. Therefore, Brazil needs to be aware of this technological evolution and make available free web platforms for integrating data, software, procedures, and methodologies for PA. The AgDataBox web platform (ADB) [18] is one of these technologies focused on DA. It is a platform that provides free computational tools for farmers, researchers, and service providers, mainly addressing PA practices. This platform integrates data, software, procedures, and methodologies to enable the growth of agricultural management in Brazil through free technologies.

Fog Computing is a highly virtualized platform that provides computing, processing, storage, and communication services between end devices and the Cloud. Processing, storage, and communication resources are similar features in both Cloud and Fog. However, Fog acts at the edge of the network, close to the devices, helping to make decisions in real-time (seconds to minutes) or even in a few days, thus performing the temporary storage of data, unlike the Cloud, which has the permanent storage of data for months and years, which are the bases for service applications that help in decision making, such as Business Intelligence (BI) [19]. However, this definition implies some characteristics that make Fog a non-trivial extension of the Cloud, as [19] presented.

Therefore, this study's objective was to construct a Smart Farm computational architecture from the development of agrometeorological stations and assembly of connectivity infrastructure and local server for receiving, storing, and sending data to the ADB-IoT (AgDataBox-IoT) application of the ADB platform.

## Method details

### Web platform AgDataBox

**Fig. 2:**
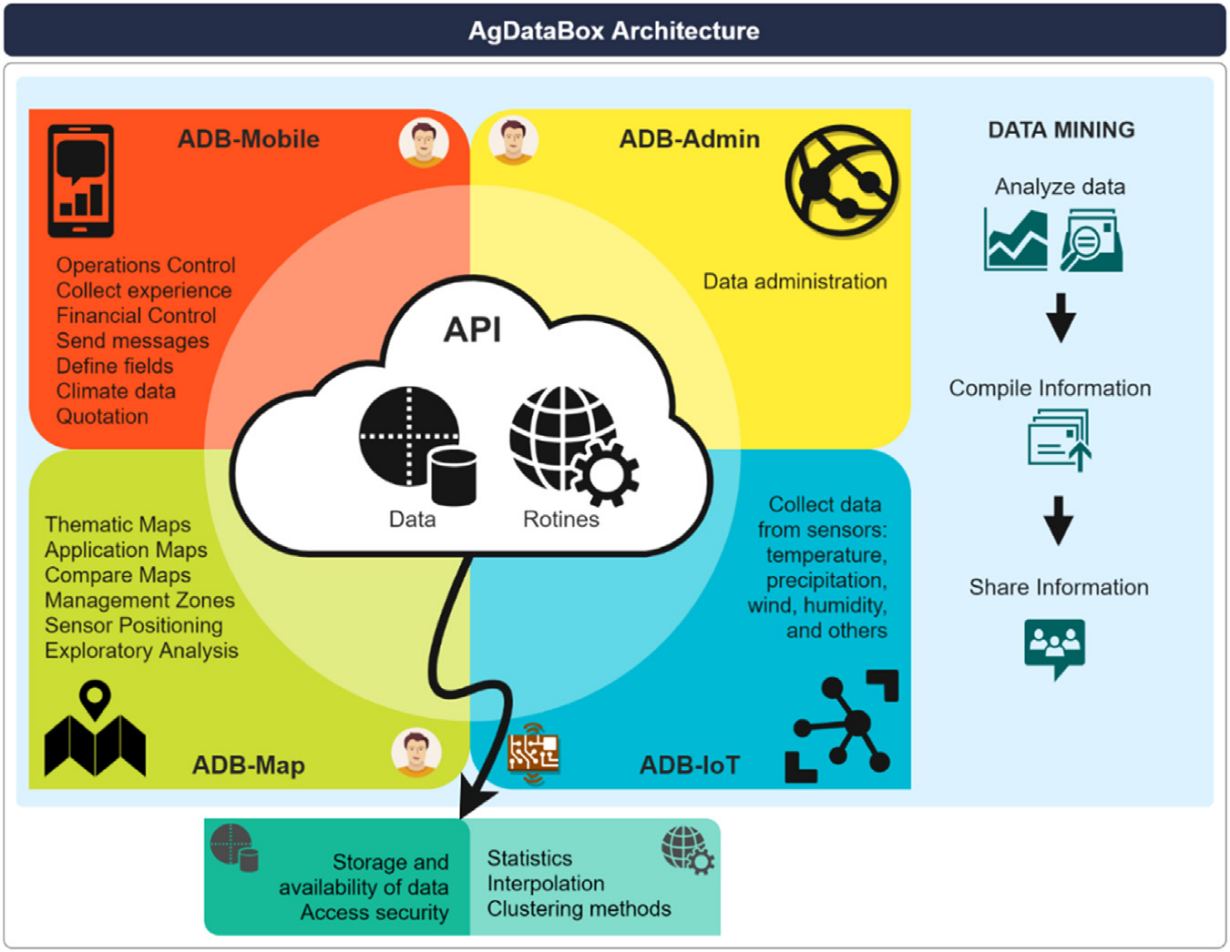
AgDataBox platform architecture design.

The AgDataBox (ADB) web platform (Fig. 2) has an Application Programming Interface (API), which consists of a set of resources accessible through the Hypertext Transfer Protocol (HTTP) for transferring requests and response messages expressed in JSON (JavaScript Object Notation) format. The ADB-API allows the interoperability of several applications in which data and processing routines are centralized. The following applications that consume ADB-API resources are under development: (1) ADB-Mobile; (2) ADB-Map; (3) ADB-Admin; (4) ADB-IoT [20,21].

### AgDataBox-IoT (ADB-IOT)

This module aims to develop networks of interconnected sensors for plant climate and water monitoring. The objective is to provide a software framework for collecting data from a sensor network installed in an agricultural area. These data are then made available in the Big Data of ADB-IoT.

### Computational architecture

**Fig. 3:**
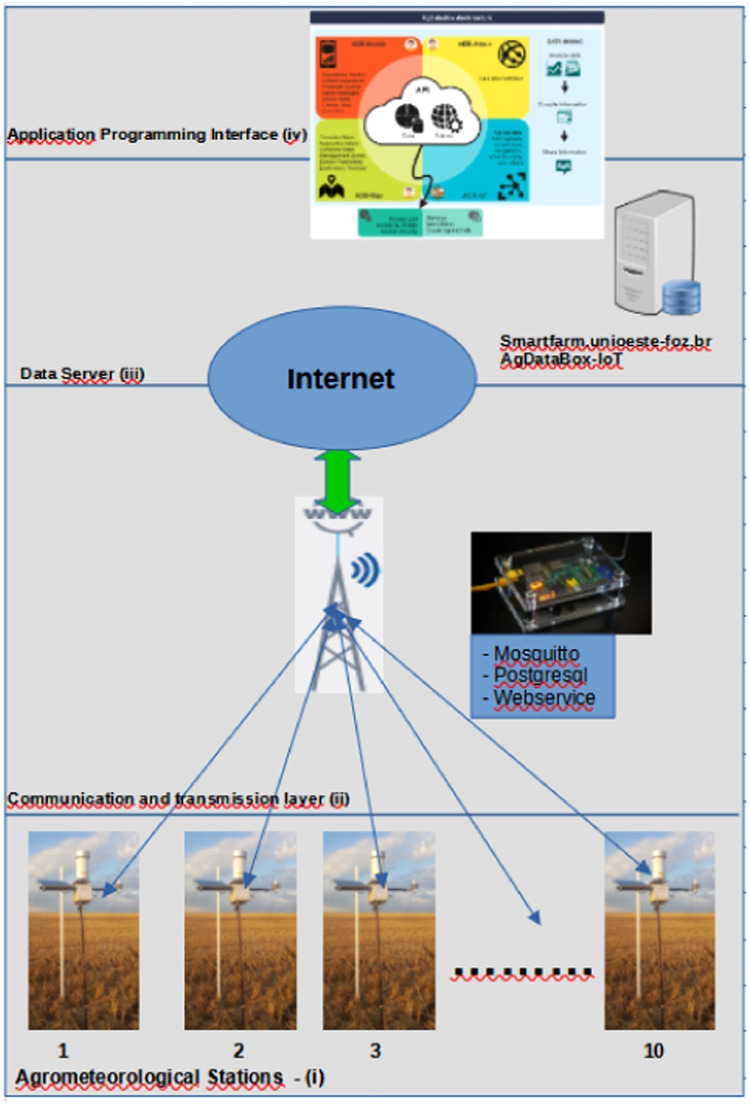
Hardware and software development layer scheme.

**Fig. 4:**
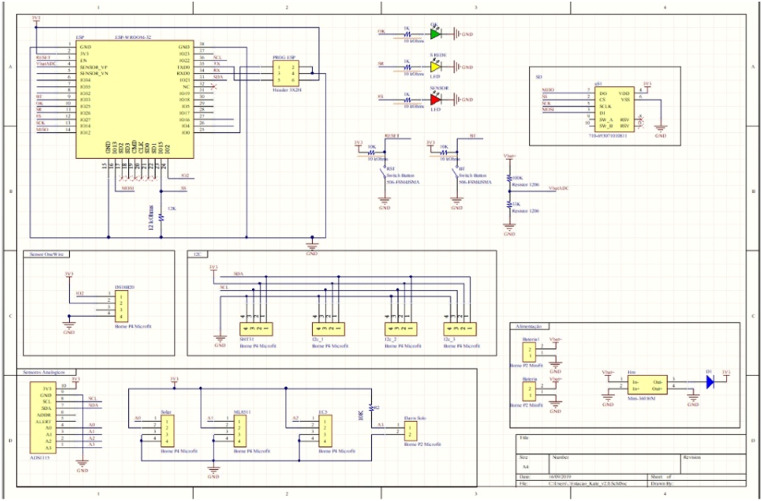
Schematic diagram of the agrometeorological station.

A computational architecture was built for the development of the ADB-IoT application, including hardware and software, which can be divided into four layers: (i) Data acquisition layer (Agrometeorological Stations – AgDataBox-IoT), (ii) Communication and transmission layer between the stations and the Data server, (iii) Data server, and (iv) API (Application Programming Interface)/AgDataBox for the data access (Fig. 4). These layers were developed independently to allow interoperability with other solutions/technologies in each layer (Fig. 3).

The communication between the stations and the local server is performed through the MQTT (Message Queuing Telemetry Transport) protocol, connected through an Access Point TP-Link TL-WA721N installed at the height of 10 m in a communication tower located on the farm. The communication between the local server and server (http://adb-iot.unioeste-foz.br:3004) is carried out through the REST (Representational State Transfer) client/server architecture by sending the data in a JSON (JavaScript Object Notation) format.

### Agrometeorogical station

#### Architecture of scalar sensor node

The agrometeorological station is composed of five main parts (Fig. 3): (i) processing, sensor data acquisition, and data transmission unit; (ii) analog-to-digital data conversion (ADC) module, which connects up to four analog sensors; (iii) I2C module, which allows data acquisition from up to four sensors with I2C output; (iv) One-Wire module, which allows data acquisition from the sensors and has a One-Wire output; and (v) MicroSD card module, which stores the collected data, being incorporated into the project for data security in case of lack of communication with the server. The station is powered by a DC source or solar battery system, supporting voltage between 3.3 V up to 28 V (Fig. 4).

### Processing and communication unit

The System on Chip ESP32 model ESP32-WROOM-32 was used in the Processing and Communication Unit, consisting of a low-cost, low-power system with Wi-Fi (802.11 b/g/n/e/I 802.11n @ 2.4 GHz up to 150 Mbit/s) and Bluetooth (v4.2 BR/EDR Bluetooth Low Energy (BLE). SoC ESP32 features a 240 MHz Tensilica Xtensa LX6 dual-core processor, Hall sensor, SD card interface, Ethernet, high SPI speed, UART, I2S, and I2C (Espressif). Wi-Fi 802.11 2.4 GHz was used for data transmission through the MQTT protocol.

### Analog interface

The environmental parameters collected by the sensors, especially those of soil moisture and some meteorological ones, are analog, operating in the voltage range of 0–3.3 V. The ADS1115, which has four analog channels with 16-bit precision and a data rate from 8 Hz to 860 Hz, was used for data acquisition from analog sensors. After conversion, the digitized data are made available through the I2C communication interface.

### I2C interface

ESP32 already has the I2C interface natively, which allows the use of sensors and other devices that communicate through the I2C interface. The developed project foresees the connection of up to four I2C devices.

### One-wire interface

Some sensors, such as the DS18B20, which measures temperature, communicate through a One-Wire interface, which has only two wires for its communication. Each device has a unique address, and the technology allows the connection of several devices/sensors on the same wire. Three DS18B20 sensors are being used in the project to measure soil temperature at different depths.

### Power unit

The agrometeorological station is powered by a solar battery that can vary between 5 V and 28 V. Considering that SoC ESP32 operates at 3.3 V, the DC-DC voltage regulator LM2596–3.3 V was used to develop the power supply module, which allows a voltage input of up to 40 V and an output of 3.3 V.

### Extra digital interfaces

**Fig. 5:**
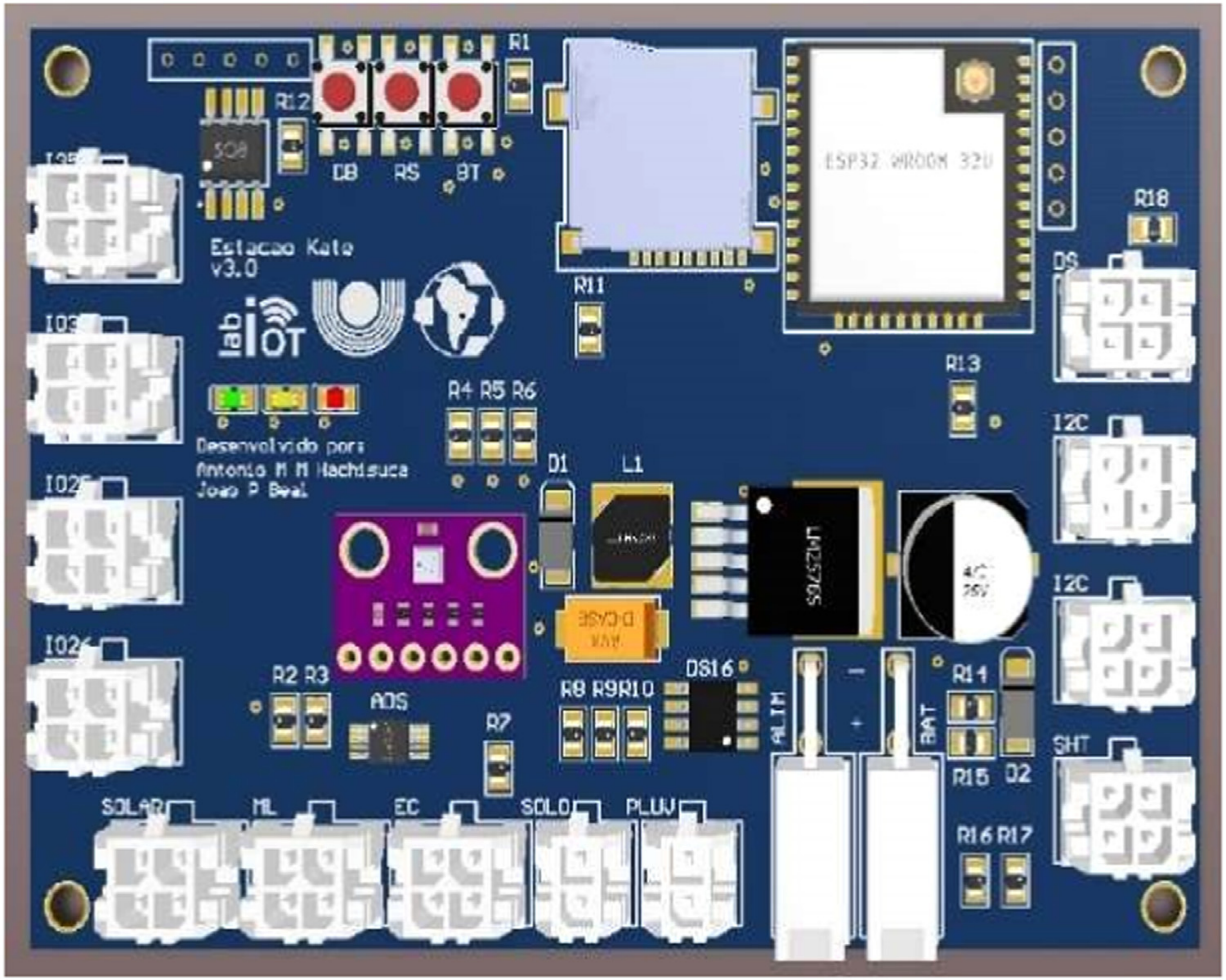
Diagram of the electronic circuit of the agrometeorological station board.

Four digital interfaces were incorporated into the project to allow the connection of other sensors or devices. Twenty PCB (Printed Circuit Board) units of the Agrometeorological Station were developed and produced (Fig. 5).

Each agrometeorological station (Kate 3.0) (Table 1) can monitor all climate parameters identified as necessary for monitoring during a growing season.

**Table 1:** Technical characterization of stations.

Item	Description
Processing	System on Chip – ESP-32 Espressif
Digital ports	04 digital channels – 3.3V
Analog ports	04 3.3 V analog channels – with 16-bit AD converter
One-Wire digital port	Capacity to connect up to 128 one-wire devices
i2C port	03 i2C ports
MicroSD slot	MicroSD card slot
Power supply	Battery between 6 - 12V
Watchdog	External watchdog
Atmospheric pressure sensor	Bosch BMP280 sensor connection slot

The climate conditions, especially air temperature, that occur throughout the crop cycle directly influenced the cultivar development, being possible to estimate when the crop development stages will occur when analyzing these events [22,23]. Climate variations such as relative humidity, air and soil temperature, rainfall, solar radiation, and photoperiod define the duration of the phenological phases of a crop, varying the number of days of the crop between regions, years, and sowing dates. The most important climate element to predict crop phenological events is the temperature, as long as there is no water deficit [24]. Several parameters were measured to monitor the microclimate of each management zone (Table 2).

**Table 2:** Measured parameters (every minute) and used sensors.

Variable	Unit	Sensors	Precision
A ir Temperature	°C	Sensirion SHT35	±0.2 oC
Air Humidity	%UR	Sensirion SHT35	±1.5%RH
Atmospheric pressure	hPA	Bosch BMP280	±1 hPa
Solar radiation	W cm^-2^	Davis K6450	±5%
Wind speed	m s^-1^	Anemômetro analógico	±1 m/s
Rainfall	mm³ h^-1^	Pluviometro Squitter	1% a 50 mm/h

SOIL			

Temperature (5 cm)	°C	DS18B20	±0.5 oC
Temperature (20 cm)	°C	DS18B20	±0.5 oC
Temperature (30 cm)	°C	DS18B20	±0.5 oC
Soil moisture	Cb	Davis 6440	1% para cada 0.5oC
Soil moisture	CVW	Decagon Ec-5	±0.03 m^3^/m^3^ CVA

The communication between the stations and the local server is performed through the MQTT (Message Queuing Telemetry Transport) protocol, being connected by an Access Point TP-Link TL-WA721N installed at a height of 10 m in a communication tower located on the farm owner's house was used for the connection between the agrometeorological stations, WIFI Access Point (AP), AgDataBox-IoT local server, and the Internet. The communication between the local server and the server (http://adb-iot.unioeste-foz.br:3004) is carried out using the REST (Representational State Transfer) client/server architecture, sending the data in a JSON (JavaScript Object Notation) format.

**Fig. 6:**
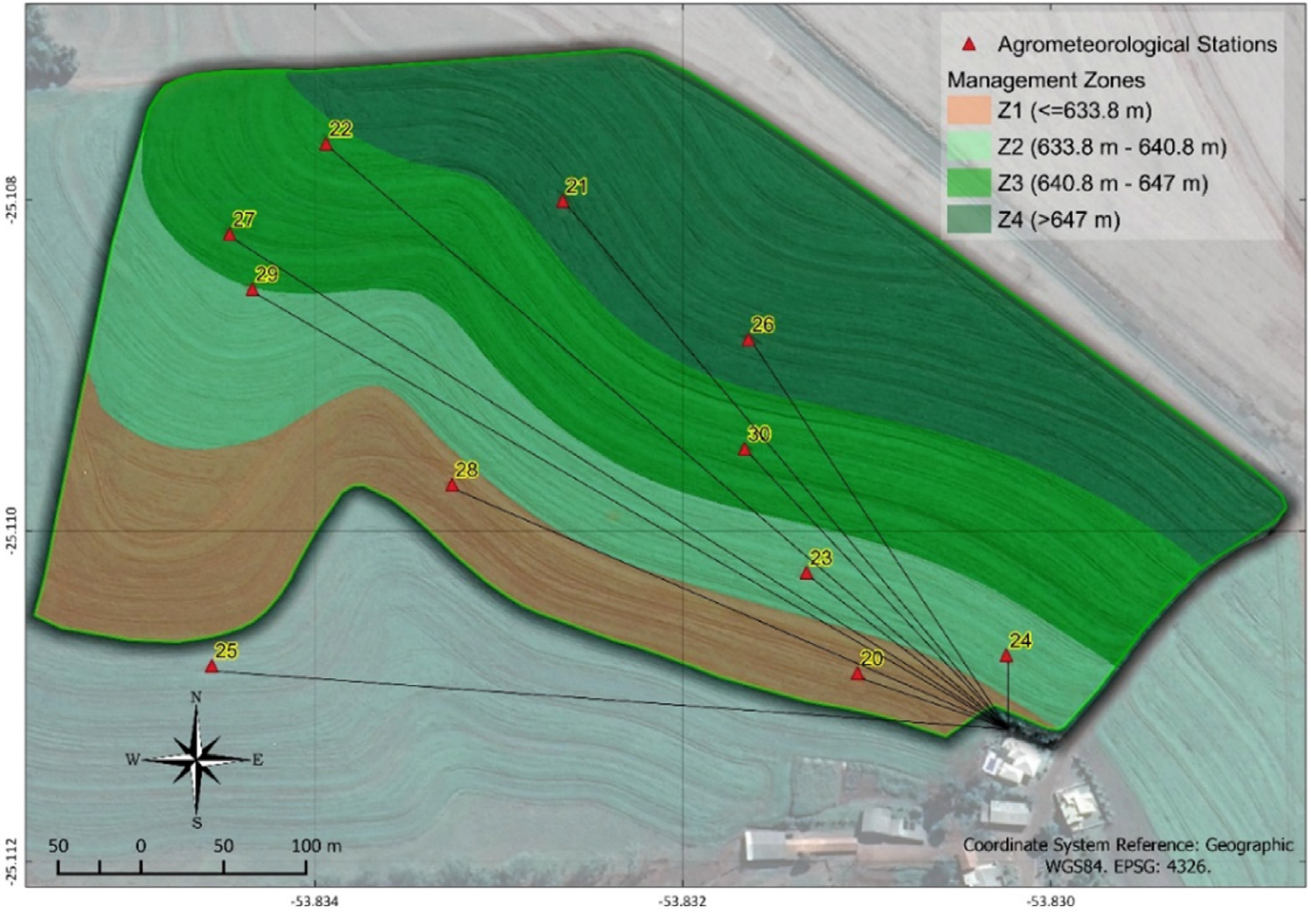
Installed network topology.

The longest distance between the Access Point and the station is approximately 500 m. The network topology is shown in Fig. 6.

### AgDataBox-IOT local server

**Fig. 7:**
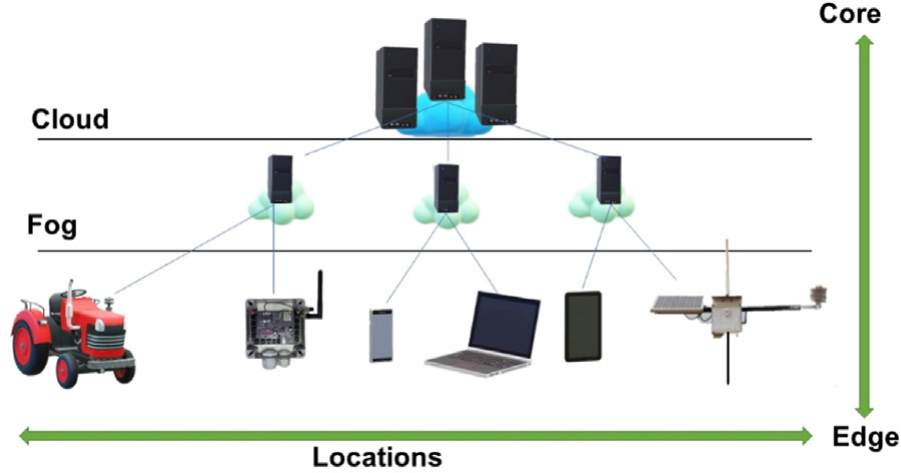
Representation of fog computing. source: adapted from Stojmenovic and Wen [26].

Considering that the availability of the Internet in rural areas can be a limiting factor for sending data to the ADB-IoT server, located in the data center of the Itaipu Technological Park (ITP), we decided to use computational resources close to IoT sensors for local data storage and pre-processing (ADB-IoT Local Server). It decreases the dependence on Internet connection as the local server stores the data locally and transmits it to the ADB-IoT server when it has an Internet link. This local server solution ensures greater reliability in data collection, allowing for further analysis for decision-making. However, edge devices cannot handle multiple IoT applications competing for their limited resources, which would result in resource contention and increase processing latency. Fog Computing (Fig. 7) integrates devices present at the edge of the network and Cloud resources, helping to overcome these limitations [25].

A Raspberry Pi 3 model B was used to develop the AgDataBox-IoT Local Server. It is a microcomputer the size of a credit card, which has a Cortex-A53 64-bit processor, with 4 cores, clock of 1.2 GHz, 1GB RAM, and 802.11n wireless standard already built-in. The Raspberry Pi was responsible for providing pre-processing, storage, and communication close to the devices, that is, locally, acting as an MQTT (Message Queuing Telemetry Transport) broker, being used the Mosca broker (MQTT) and implemented in Node.js.

The MySQL database configured on the Raspberry Pi was used to store the data locally. The MQTT protocol was used through a wireless network for communication between the stations and the broker. It was chosen because it is a light and flexible protocol, with low energy consumption, besides being prominent in several IoT applications.

Two APIs were developed for the server also in Node.js, both working in the cloud (adb-iot.unioeste-foz.br). The first one is for receiving data aiming to provide communication, in addition to processing and storing the information coming from the fog, and the other to manipulate the information from the agrometeorological stations, their sensors, and readings of these sensors, besides making all this information available to the user.

**Fig. 8:**
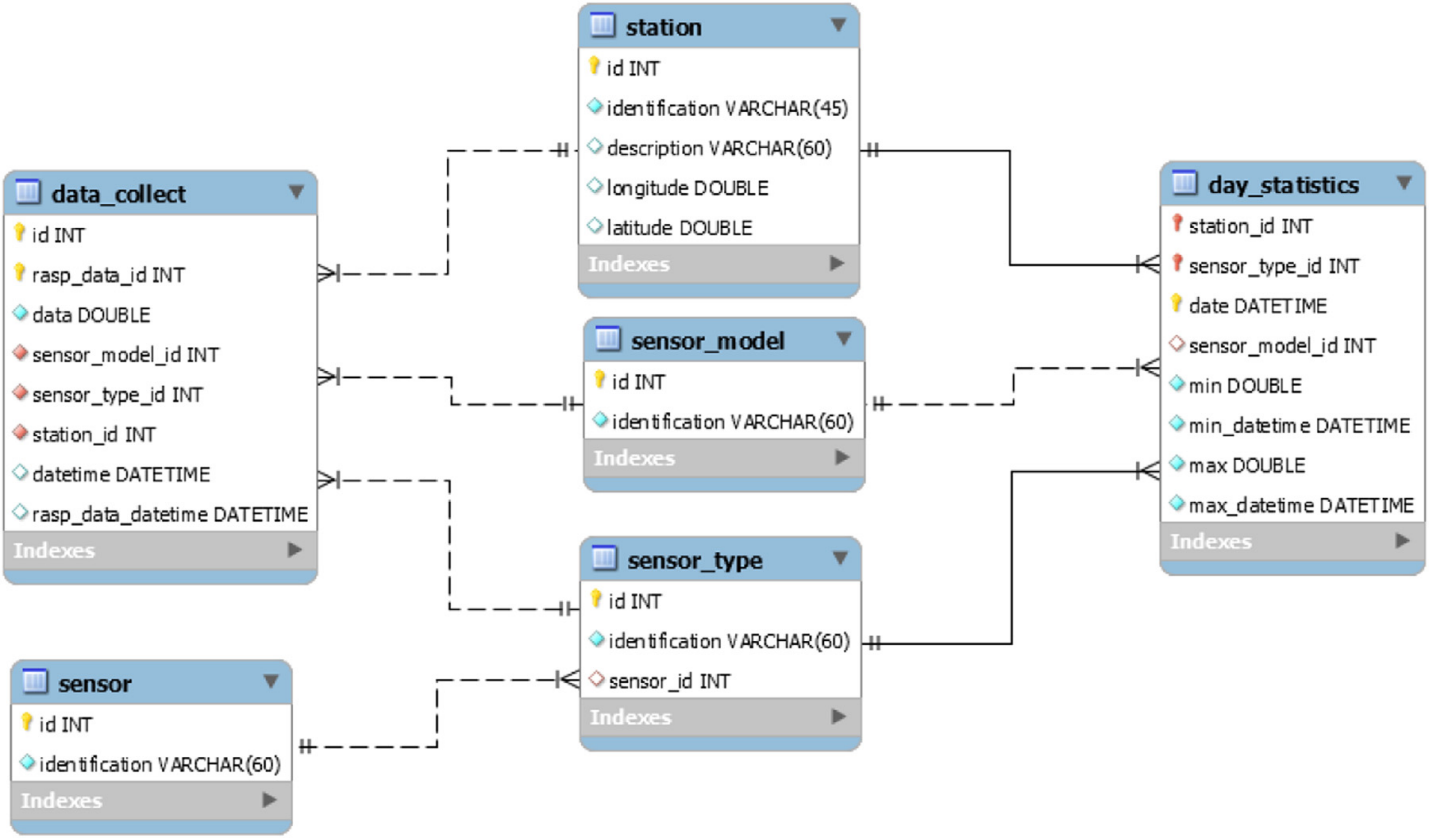
Flowchart of the entity-relationship database model.

For this, a Database was defined with the following Entity-Relationship model (Fig. 8).

The sending of data from the stations is carried out through port 3000 and REST/JSON protocol, in which the sensor data and acquisition date and time are sent. The API includes the date and time the data was received. Thus, the delay between receiving data from the stations and the time for storage on the server can be evaluated. Also, it is possible to monitor if there was a drop in the farm's internet connection and the time required for data recovery/synchronism.

### API (Application programming interface)/agdatabox

An API was developed as a form of integration with the AgDataBox platform, especially the AgDataBox-Admin module, operating on port 3001, allowing the register of new stations, sensors, and access to data stored on the AgDataBox-IoT Server (adb-iot.unioeste-foz.br).

The methods above allow accessing the data stored on the smartfarm.unioeste-foz.br server to consult the mean, maximum, and minimum temperature of station 25 on November 8, 2020, for example. The command and result are shown in Chart 1.

**Chart 1:** Route and result for one-day temperature summary access.

http://adb-iot.unioeste-foz.br:3002/estacoes/25/tipo_sensores/1/resumo_dia?&data=2020–11–08 { "resultado": { "estacao": { "id": 25, "nome": "Estacao 25 Aldo" }, "data_dia": "2020–11–08T03:00:00.000Z", "tipo_sensor": {"id": 1,"nome": "Temperatura do Ar"}, "media_dia": 27.40963746223565, "min": 20.41, "data_hora_min": "2020–11–08T05:23:40–03:00″, "max": 35.65, "data_hora_max": "2020–11–08T11:55:16–03:00″, "graus_dia": 20.03 }, "erro": null }

### Data analysis

The necessary procedures and calculations were carried out in an electronic spreadsheet. The data were tabulated in ten-day periods. Subsequently, a descriptive analysis of the data collected from agrometeorological stations was carried out. The parameters mean, standard deviation and coefficient of variation were calculated for the 10 stations installed in the area and each delimited management zone.

### Study area

**Fig. 9:**
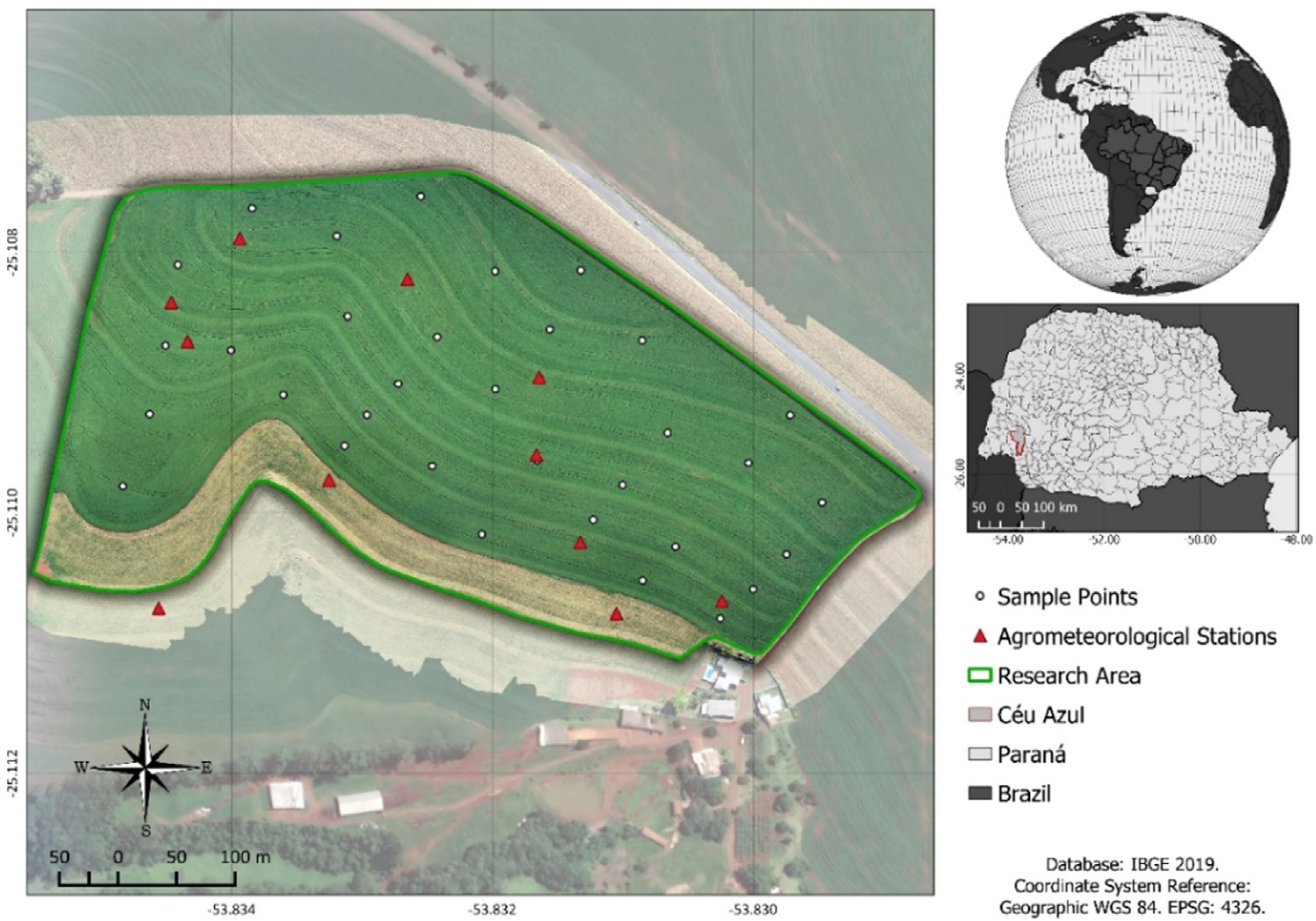
Location of the study area. Scenario analyses.

Eleven agrometeorological stations (Fig. 9) were installed in a 17.9-ha area located in the municipality of Céu Azul-PR, with central coordinates of 25°07′08.50″ S and 53°50′10.51″ W. The soil in the region is classified as a typic Dystroferric Red Latosol [27], and the field has been cultivated under a no-tillage system for at least 15 years, with commercial soybean, corn, and wheat crops are grown in succession. The region has a subtropical climate (Cfa), with a mean temperature in the coldest month below 18 °C (mesothermal) and a mean temperature in the hottest month above 22 °C, in addition to hot summers, infrequent frosts, and a tendency for rain concentration in the summer months, but with no defined dry season.

**Fig. 10:**
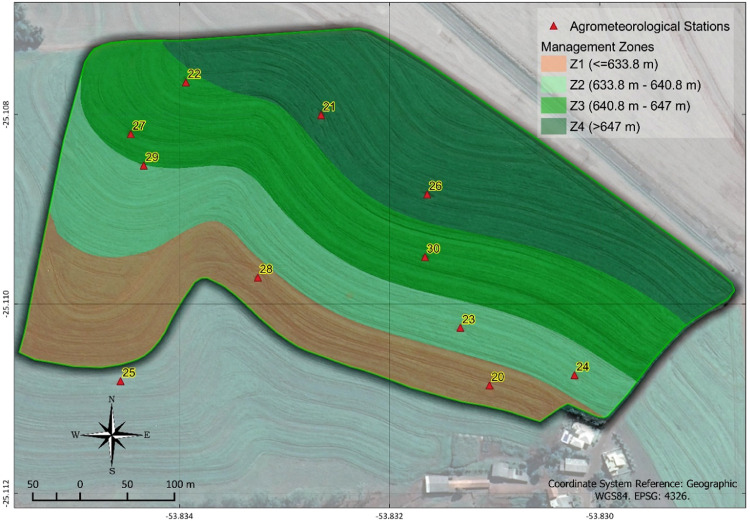
Delimitation of management zones 1 (Z1), 2 (Z2), 3 (Z3), and 4 (Z4) in the experimental area.

The location determined for each station's installation (Fig. 10) was determined using the AgDataBox application – Sensor from the ADB platform Bazzi et al. [28] through elevation data. The Fuzzy C-Means algorithm allowed determining the ideal locations for at least two stations in each of the four zones. The division into four management zones was found in Schenatto et al. [29], using altitude (m) and soil penetration resistance 0–0.1 m (MPa).

### Agrometeorological station architecture

**Fig. 11:**
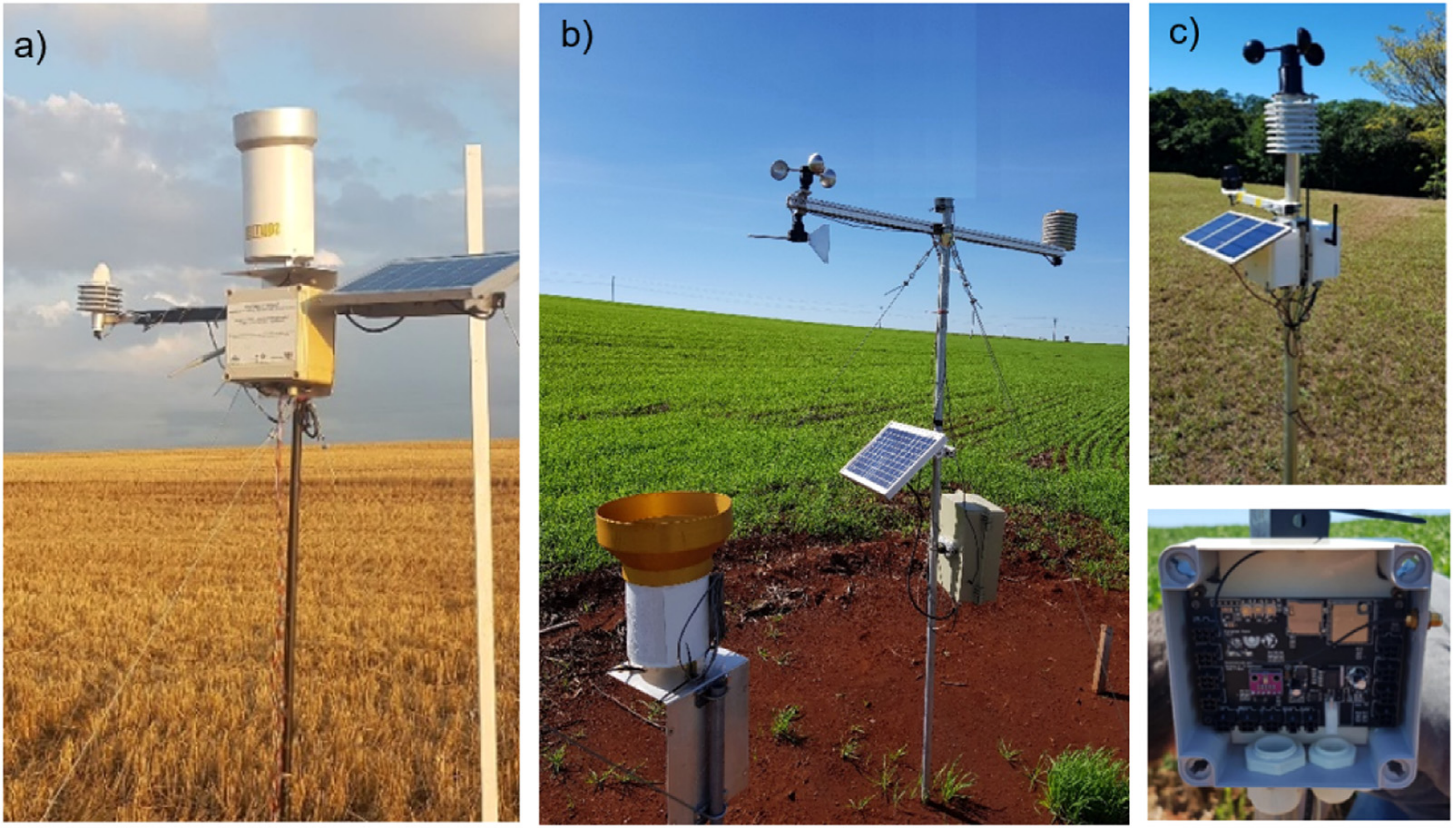
Architecture of agrometeorological stations in (a) 2018, (b) 2019, and (c) 2020.

Fig. 11 shows the architectures of the agrometeorological stations developed throughout the project. An advance was observed in the construction of stations between 2018 and 2020, as the station installed in 2018 had a simpler architecture (Fig. 11a). An electronic board for data collection was developed based on the existing Squitter rain gauge and using the Wemos mini d1 module, which has an Espressif ESP8266 System on Chip (SoC). However, despite meeting the requirements for the collection of climate data, it presented reading problems during field tests, especially in the reading of rainfall, as the rain gauge was installed at the top of the station, which was influenced by the wind, resulting in erroneous readings when wind gusts occurred, besides and poor contact at the sensor connectors. Improvements were made in the second version of the electronic design, which was developed in 2019 (Fig. 11b), with changes in the connector model to ensure better reliability and reduce failures in sensor readings. It was also installed a rain gauge on an exclusive basis, resulting in a more reliable reading, as the wind gust less influences it. However, similarly to the 2018 version, its installation in the field required a team of at least 2–3 people and the use of stakes. Finally, several improvements were made in the 2020 version (Fig. 11c), with the replacement of SoC ESP8266 by a higher- processing-capacity SoC ESP32, the development of a new electronic project (Fig. 7) using the components in SMD (Surface Mounted Device) format, making the project more reliable and professional (product), the use of more compact solar panel and batteries, the ability to install several sensors (Table 2), and the mechanical design, which allows its installation by a single person without the need to install stakes in the station.

Table 3 shows the count, sum, and mean data acquired by the stations from June 2019 to December 2020. It evidences one of the significant problems when working with data measured in agrometeorological stations, which consists of the data incompleteness due to equipment failure in case of an automated system or adverse conditions, which may damage the station [30].

**Table 3:** Overall characteristics of data counting of the stations in the period from June 1, 2019, to December 27, 2020.

Station	Total days with data	Average number per day
20	547	1232.47
21	383	1208.04
22	499	1342.57
23	496	1038,23
24	530	1228.18
25	421	2536.69
26	362	1226.90
27	340	1267.97
28	378	1012.72
29	436	1273.19
30	428	1085.25

Twenty-eight of 575 days of analysis had no data acquisition by any of the stations. Moreover, station 20 obtained data collection for 547 days. Considering that the stations did not always stop working simultaneously, the number of days without information was even smaller. Using more than one station in close regions minimizes this data loss effect and provides data with greater consistency, ensuring continuous data collection, without acquisition failures due to station failure, disregarding external factors such as power outages or the fall of stations due to gales.

**Fig. 12:**
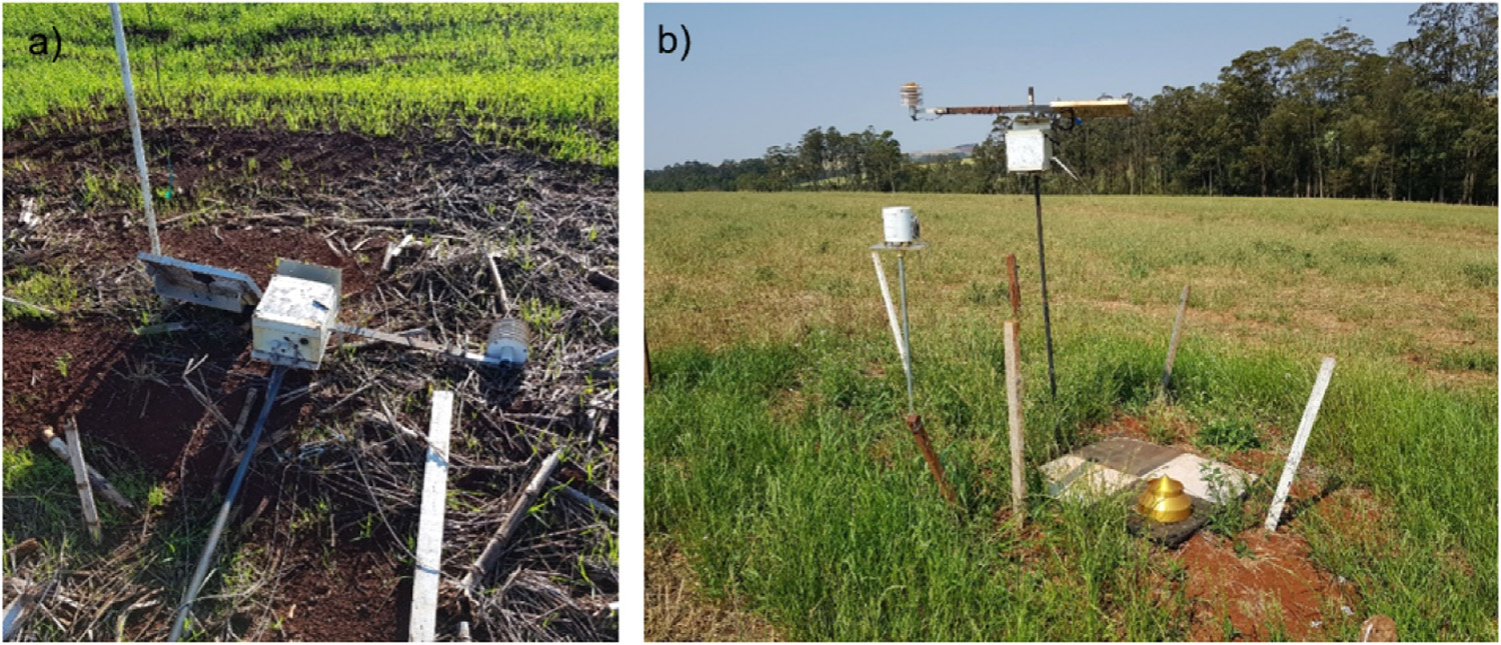
Illustration of stations with data collection problems: (a) wind-damaged station and (b) part of the sensors carried by the wind.

Some of the stations had a lower total of days with data, showing failures in data collection. In this sense, station 27 presented 340 days with data, possibly due to problems. Fig. 12 shows some of the events that interfered with the total number of days with data from some stations.

### AgDataBox-IoT local service

**Fig. 13:**
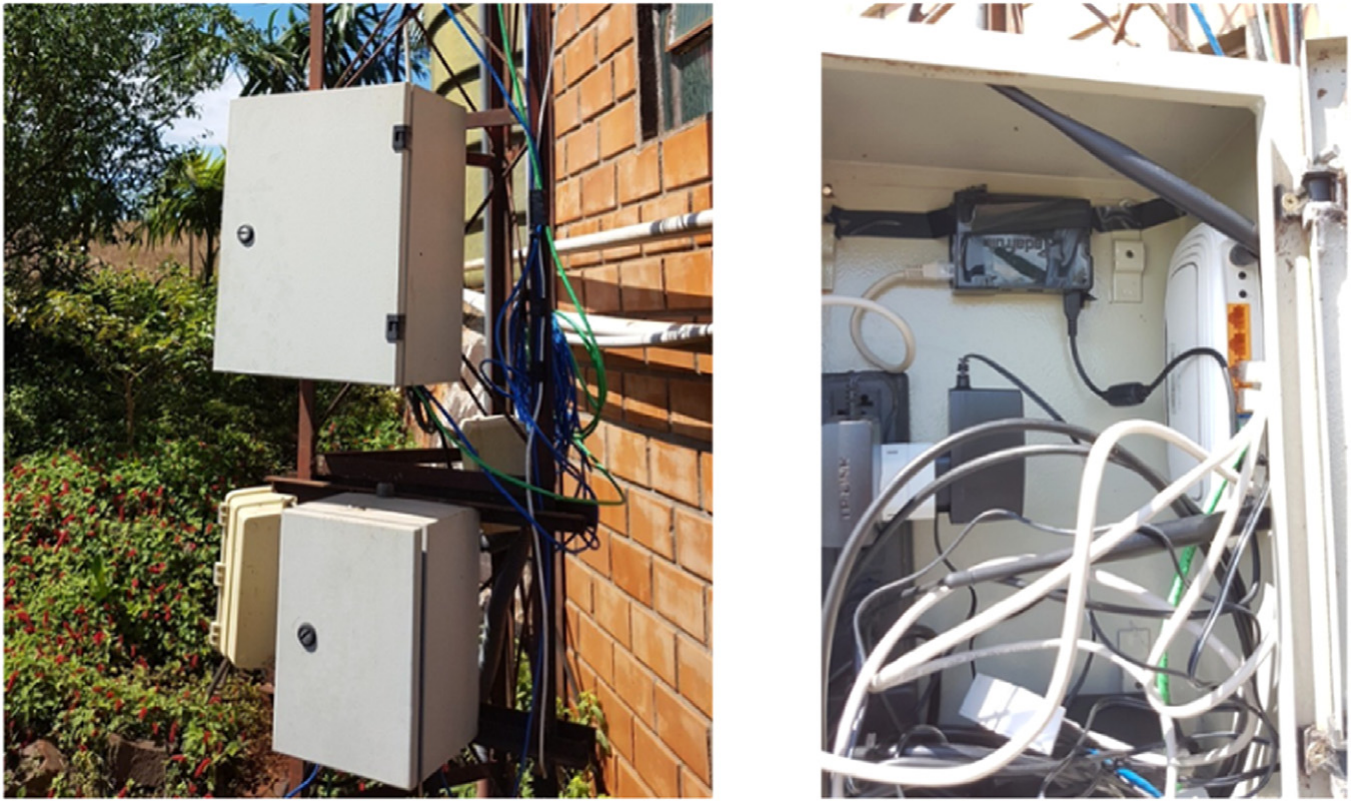
Location of the ADB-IoT local server installation.

The local server was installed at the base of the communication tower on the farm (Fig. 13) and connected to the farm's Internet network and the Access Point, making the Internet available to the stations installed in the farm's plot.

**Fig. 14:**
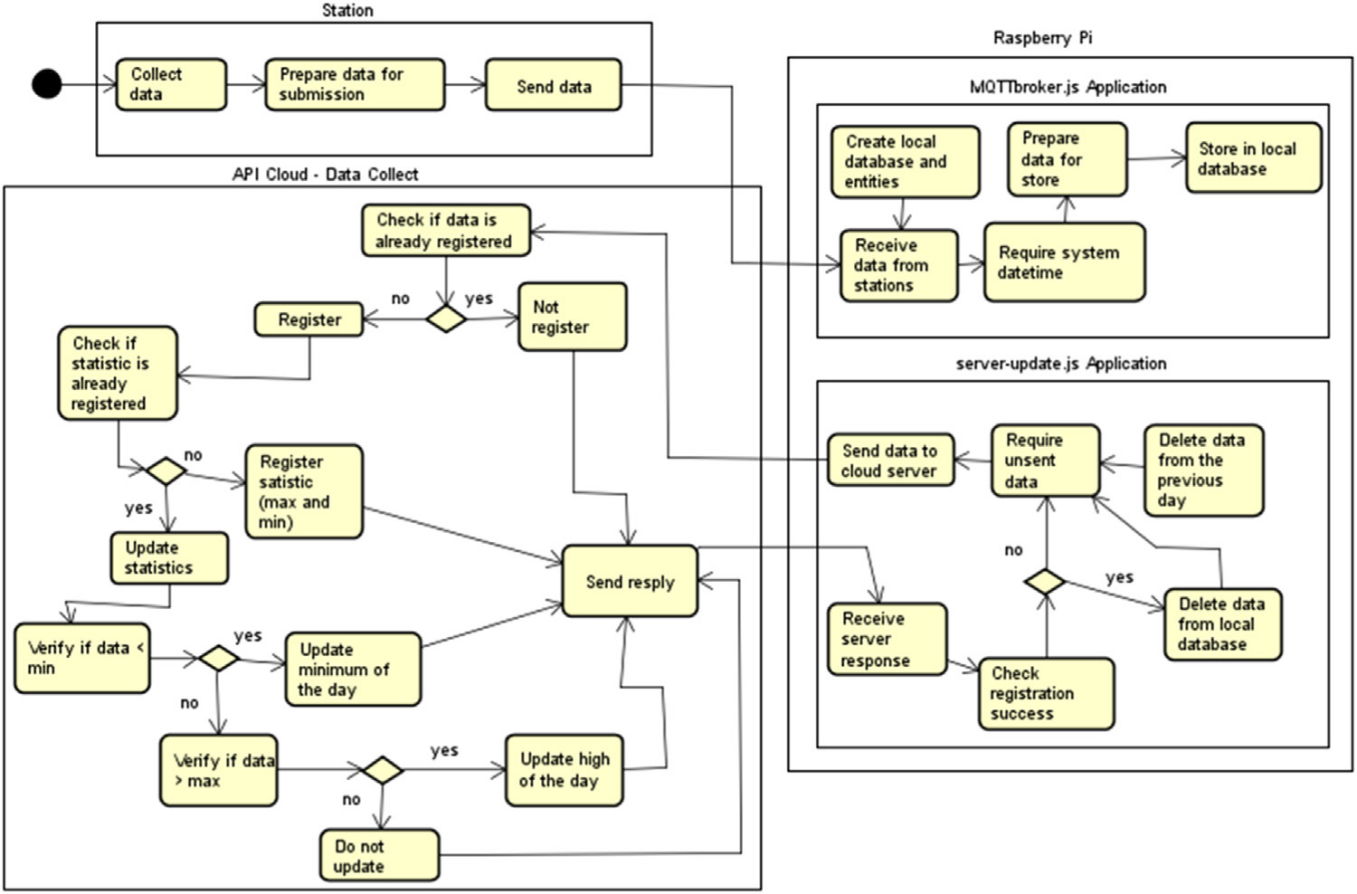
Illustrative communication flowchart between stations, local server, and the server located in the PTI data center.

Fig. 14 shows the illustrative communicatin flowchart between the local server stations and the server located in the ITP data center. First, the stations send the acquired data via MQTT protocol to the ADB-IoT local server, which receives, processes, and stores them in the local database (MySQL). Then, after storing the data locally, the ADB-IoT local server connects via REST protocol to the ADB-IoT server through an API developed for this purpose, sending the data to the server after performing the authentication and verification that the data had already been sent and deleting them from the local database after receiving the confirmation of success. This process is repeated every time the local server receives some data from a station.

**Fig. 15:**
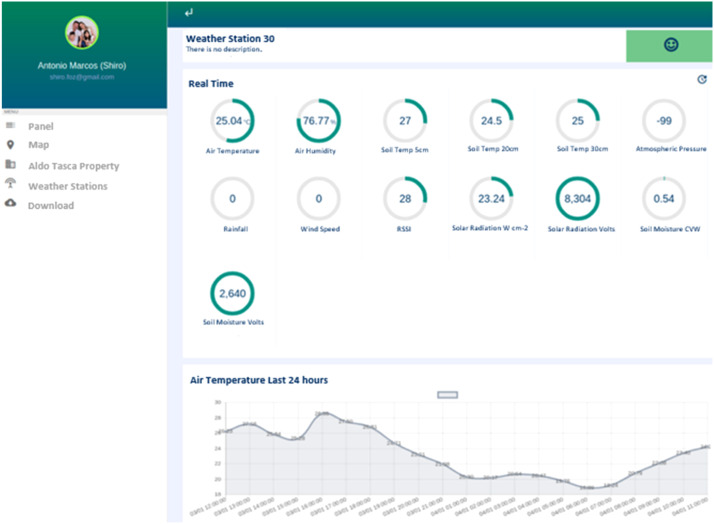
AgDataBox-IoT user interface for viewing weather variables.

Fig. 15 shows one of the user interfaces of the AgDataBox-IoT module, with air temperature data of the last 24 h for a given station. Clicking on the parameter shown at the top allows changing the graph presented at the bottom of the screen to another parameter.

**Fig. 16:**
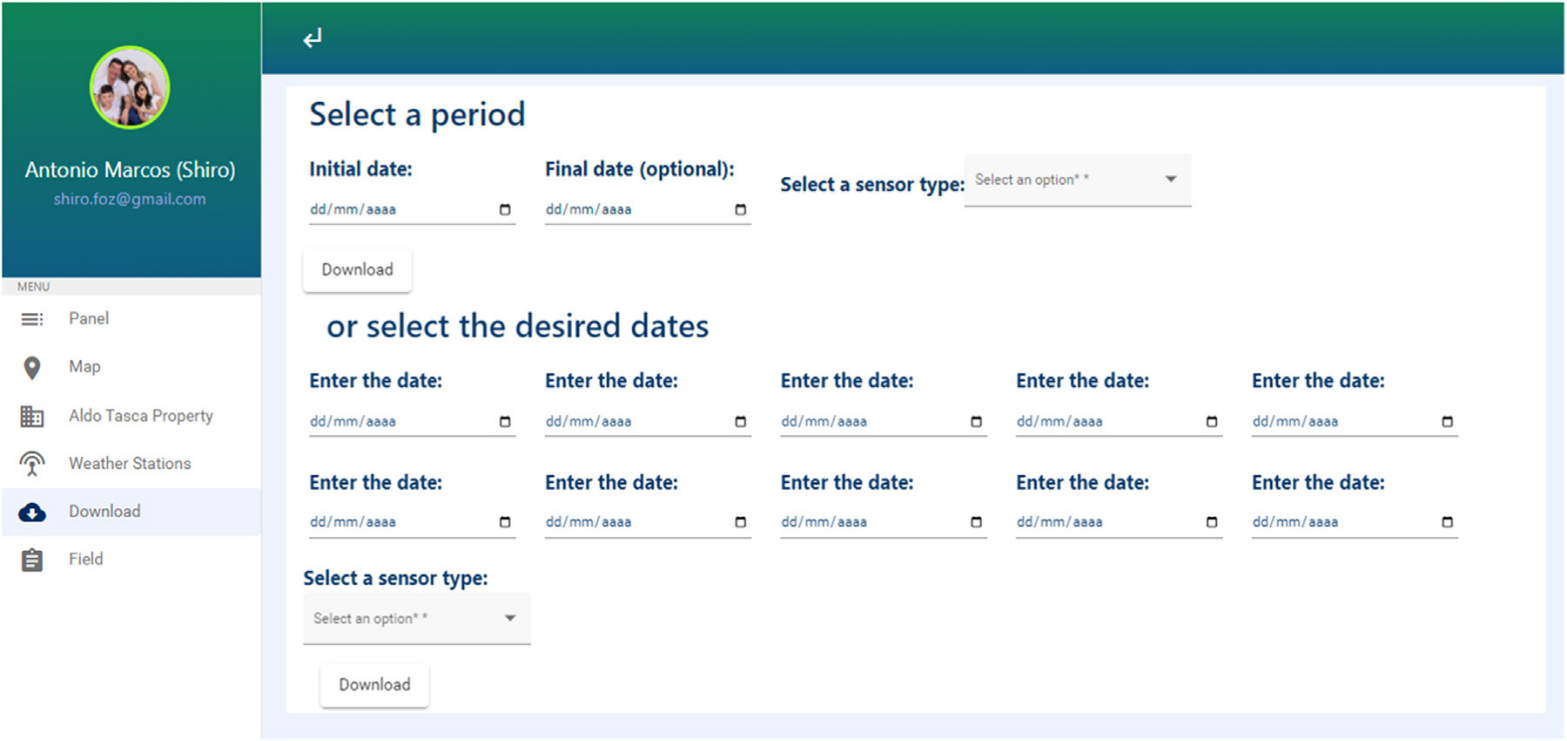
ADB user Interface for filtering data by the period of interest.

Users can access the data through the Download icon, located on the left corner of the screen (Fig. 16), allowing data to be downloaded in XLS format between periods of a particular parameter or dates from all stations. These two options allow the researcher to work with data from the entire period of a growing season or some specific dates, according to the phenological stage of the crop.

File generation functionality is being implemented in a compatible format as a sample grid layer in AgDataBox-Map, facilitating the use of climate data in the delineation of Management Zones.

An API was developed to integrate with the ADB platform, especially the ADB-Admin application, operating on port 3002, registering new stations and sensors, and accessing data stored on the ADB-IoT server (adb-iot.unioestefoz.br:3002).

### AgDataBox-IOT-API (ADB-IOT-API)

The API located at http://adb-iot.unioeste-foz.br:3002/[+rota] allows the interoperability between systems; that is, any application or web service can access the data from stations, allowing the integration of climate data by other platforms or applications. The authentication module, which will guarantee that only authorized users will access the API, is in the final phase of implementation. This API allows the data collected from the stations and the calculations/methods implemented from the data to be consulted.

### Station data analysis

The relative air humidity is an essential parameter in agriculture, as it assists in determining predictions of pest and disease infestation. In general, periods of high relative humidity favor disease development [31]. In addition, this measurement helps to determine the appropriate time for pesticide application, such as the ideal relative humidity condition above 60% for the spraying operation [32].

**Fig. 17:**
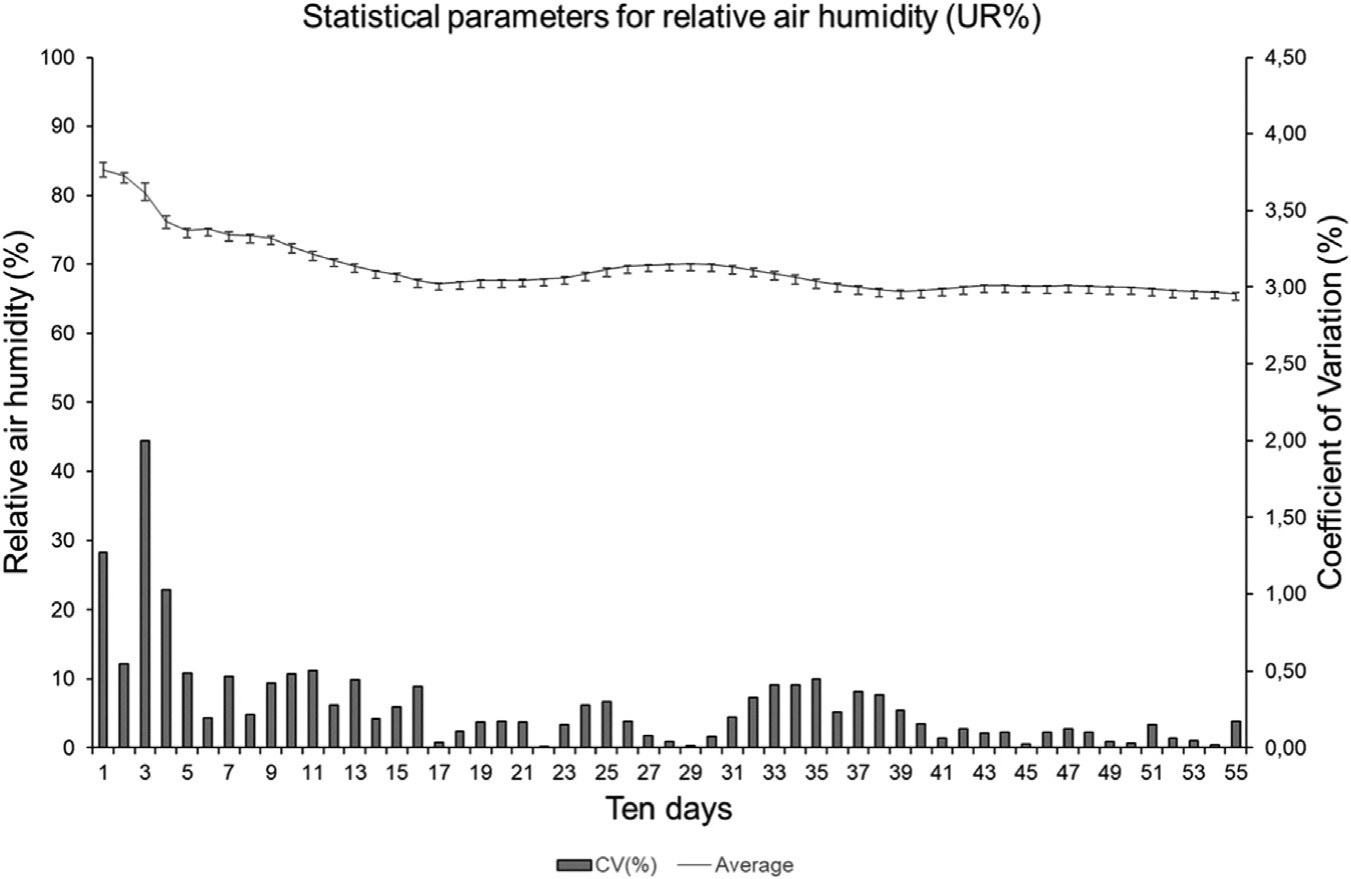
Statistical parameters of agrometeorological stations regarding the relative air humidity.

The ten-day measurements mean for all stations installed in the area (Fig. 17) presented relative air humidity values ranging from 70 to 90%. The coefficient of variation (CV) showed low variability in the data collected by the stations, with a maximum of 2%, but most of the observed data presented CV values below 1%. The standard deviation bars show small distances, indicating a slight deviation of the values relative to the mean, demonstrating that the measurements were efficient. However, the data that presented higher CV values are observed at the beginning of the measurement cycle (third ten-day period), probably due to the improvement carried out in the stations during the tests.

**Fig. 18:**
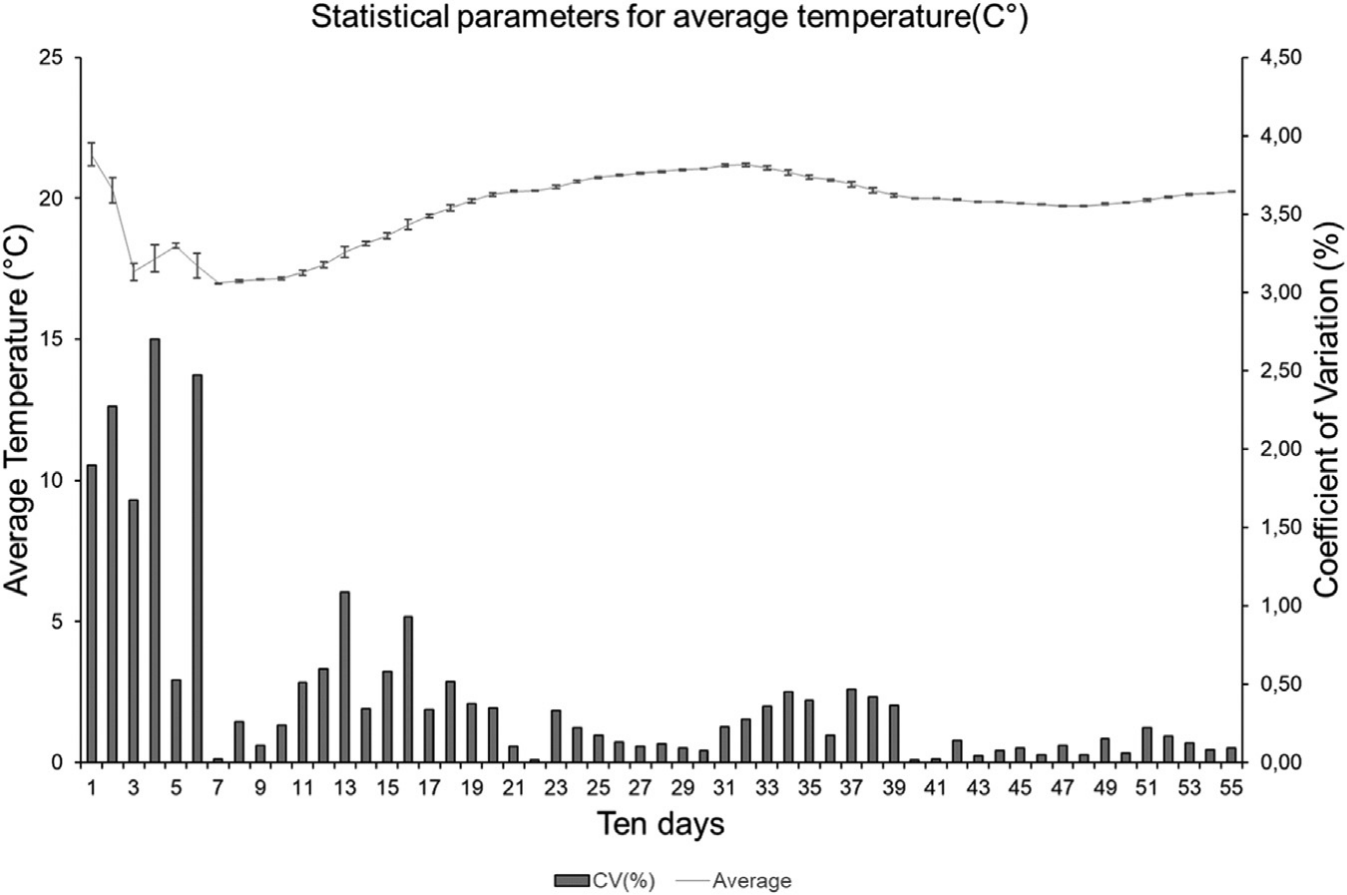
Statistical parameters of agrometeorological stations regarding average temperature.

The ten-day stations mean temperature over the periods (Fig. 18) was also measured and evaluated, with values ranging from 16 to 24 °C. The CV for this parameter is below 3.5% throughout the measurement period, with most data showing CV below 1.5%. The highest CV values are observed at the beginning of the measurement cycle (sixth ten-day period), as observed for the relative humidity measurements.

Temperature is one of the main factors influencing crop development, and its association with relative air humidity has a combined effect; that is, humidity helps the crop retain water content, but high temperatures make this process difficult [33]. Moreover, knowing the temperature at the time of pesticide application is crucial for success in reaching the target. Temperatures above 30 °C can induce plants to stress, making pesticide absorption and translocation difficult, while temperatures lower than 10 °C also lead to losses regarding application efficiency [32].

### Station data analysis by management zone

**Fig. 19:**
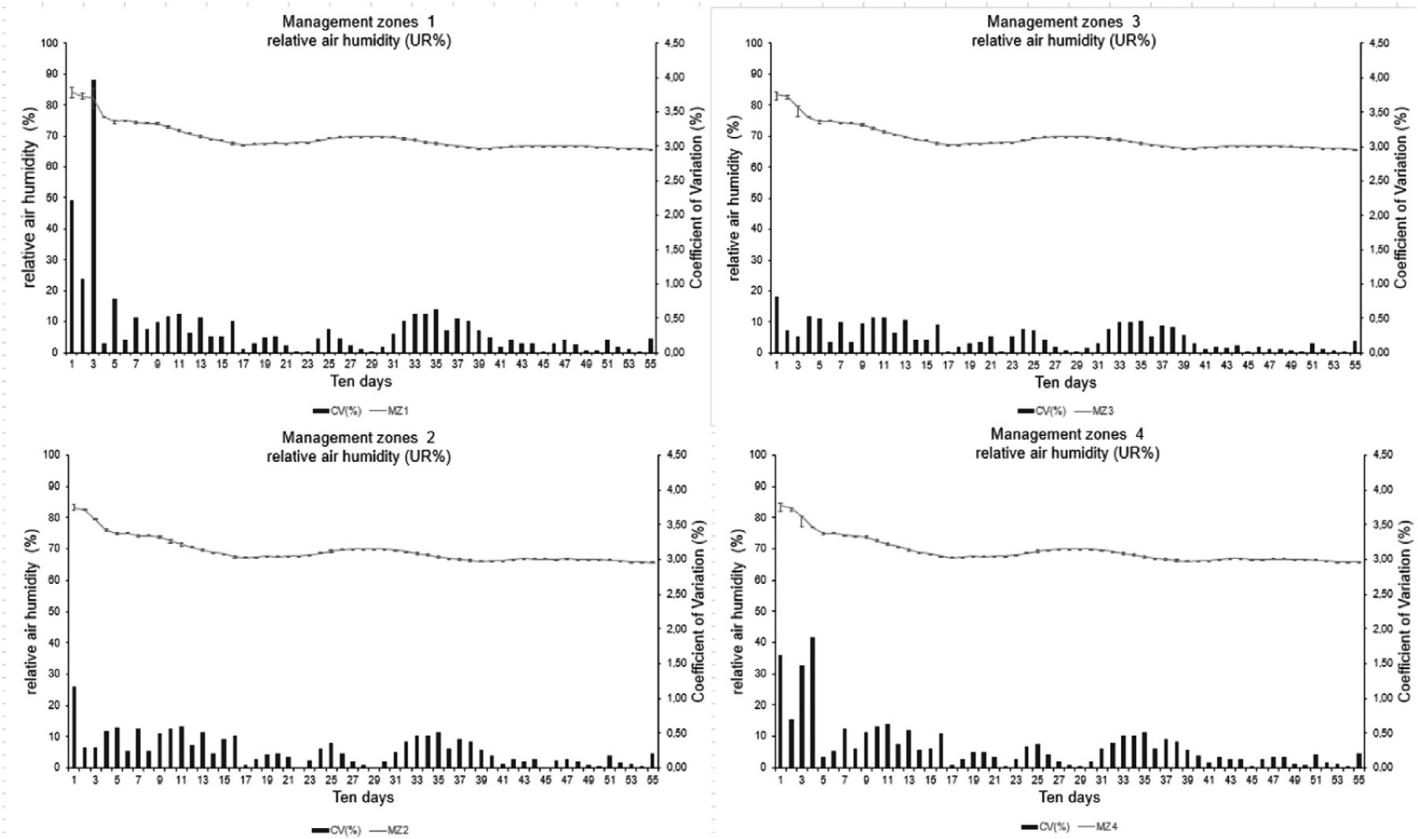
Statistical parameters of the variable relative air humidity by management zone (MZ).

**Fig. 20:**
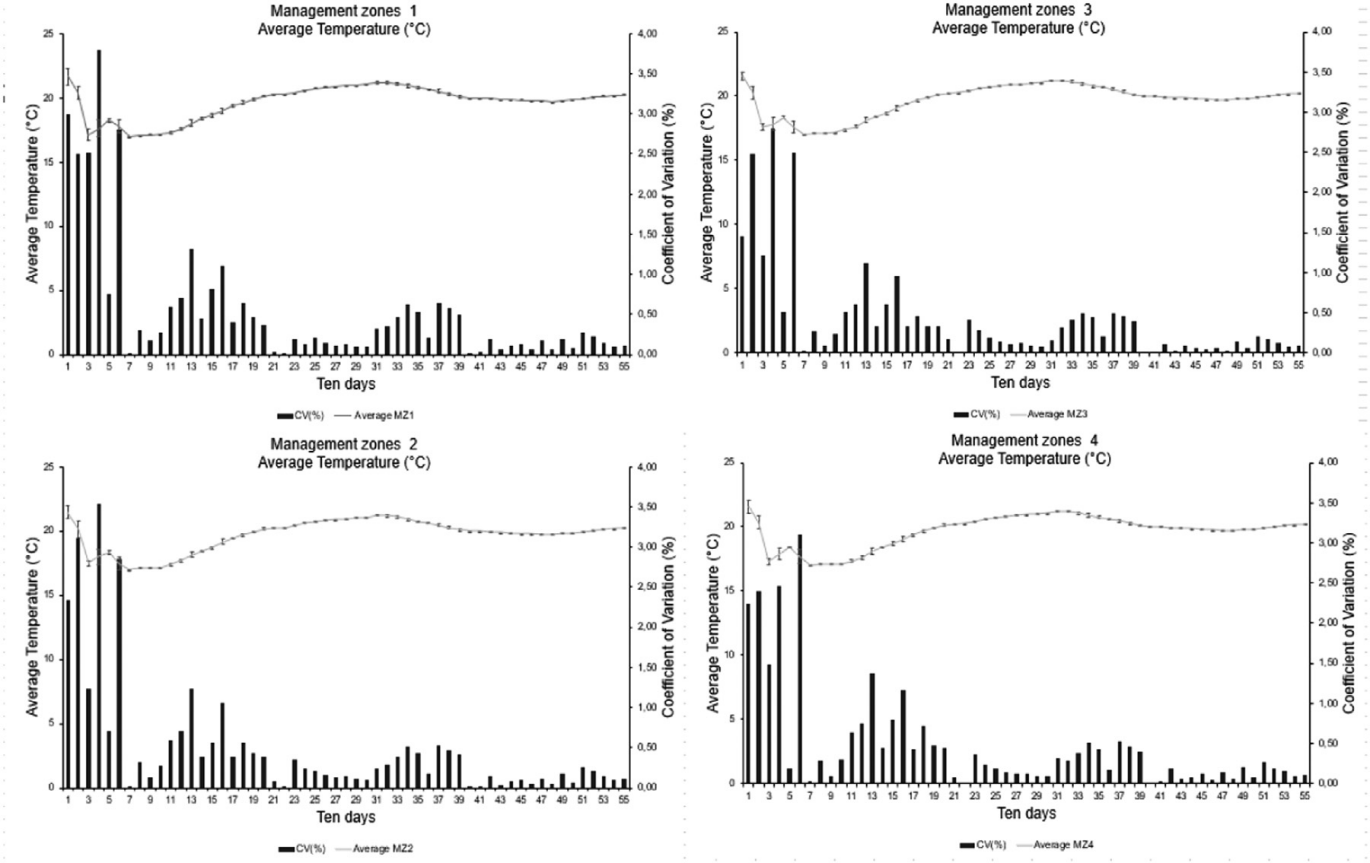
Statistical parameters of the variable mean temperature by management zone (MZ).

Management zones (MZs) are delimited sub-areas within a field, allowing for uniform individual management. They are based on spatial studies of soil and plant variables and used for fertilizer application, soil management, and irrigation on a localized scale after their establishment [34,35]. Moreover, MZs can be influenced by several factors [35], making the spatial study of climate conditions necessary to test their degree of influence on other variables and verify their variability in a given plot. Figs. 19 and 20 show the mean relative humidity and temperature of the variables under study, respectively.

The mean relative humidity values of MZs show similar behavior to the curves, evidencing a low variability in the area. Furthermore, only MZ1 presented a CV value close to 4%. It is due to a data collection failure in one of the stations in the third ten-day period. The other MZs showed CV values lower than 2%, indicating good data uniformity.

**Fig. 21:**
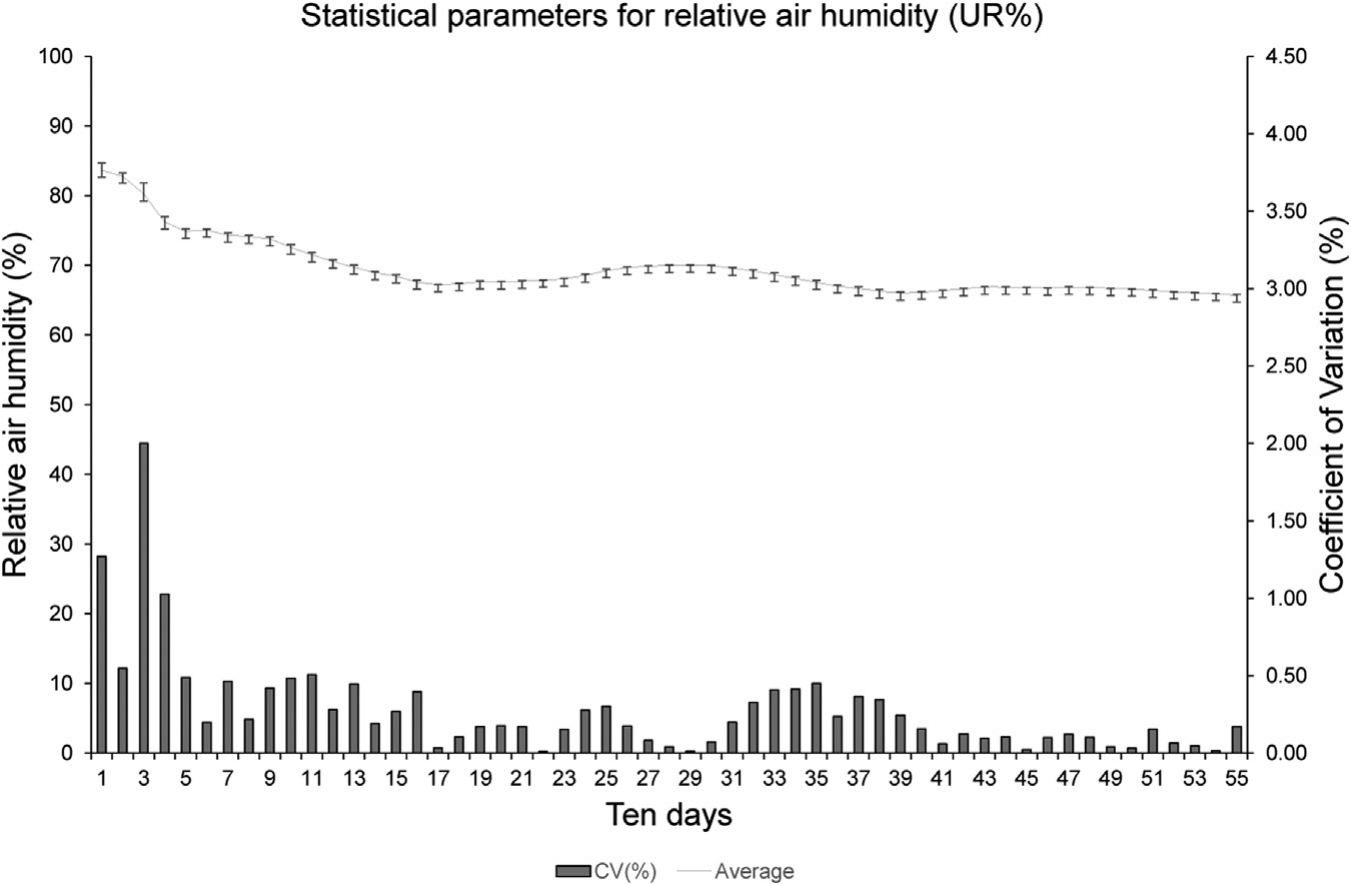
Statistical parameters of the variable mean relative humidity by management zone (MZ).

The mean temperature curve of each MZ had similar characteristics, indicating a little spatial variability in the area, as observed for relative humidity (Fig. 21). The standard deviation bars on the graphs show that the measurements are generally close to the mean. The CV values presented from the seventh ten-day period are lower than 1.5%, indicating higher stability in the measurements from this period. This behavior is due to data collection failures in the initial measurement cycle. This effect indicates that the installation of several agrometeorological stations did not present significant variability, demonstrating the possibility of monitoring the area with only one station. However, the area has a relatively small extension (15 ha) and does not have a steep slope, with the mean elevation of MZ1 being 633 m (altitude), which is the lowest portion of the area, with 647 m referring to MZ4.

## Conclusion and future work

The computational architecture developed, and the low-cost stations made it possible to acquire, transmit, store and process data without significant losses, except in sporadic events of severe weather events.

The sensors used, for the most part, already reading their data through the I2C digital interface, showed outstanding durability and accuracy in the readings since none of them had defects during the project, and one of the factors that contributed was the self-calibration capacity of these sensors, which are of the state-of-the-art, reducing the need for laboratory calibration.

The installed stations allowed the collection of data with low CV, indicating the accuracy of the temperature sensors used, and in the 15 ha experiment area, there was no significant difference in temperature between the Management Zones. However, in the first ten days, they presented the highest CV values, which decreased over time, showing the improvement of the stations.

The AgDataBox-IoT module needs new adjustments for the data filtering to facilitate the use of data by the user. It is necessary to add filters regarding the date, the data analysis period (ten-day periods, for example), and the amount of data collected daily.

It is suggested that the integration of the AgDataBox-IoT and AgDataBox-MAP modules enables data analysis from both platforms without the need for download and upload, besides allowing downloading files in shapefile format.

Studying data communication alternatives between stations and the local server, such as Lorawan, would allow broader coverage. For example, using a 2.4 GHz WIFI technology, it was possible to reach a radius of 600 m between the station and the communication tower, but it is expected that with LoraWan, this range could be 10 km or more.

## Ethics statements

The method discussed in this scientific article did not involve studies with humans, animals, or the use of platform data. All steps were conducted exclusively on an experimental research farm.

## CRediT authorship contribution statement

**Antonio Marcos Massao Hachisuca:** Conceptualization, Methodology, Software, Writing – original draft, Visualization. **Eduardo Godoy de Souza:** Writing – review & editing, Supervision, Visualization. **Wendel Kaian Mendonça Oliveira:** Methodology, Writing – review & editing, Writing – original draft. **Claudio Leones Bazzi:** Methodology, Software, Visualization. **Diandra Ganascini Donato:** Investigation, Writing – review & editing. **Isaque de Souza Mendes:** Investigation, Writing – review & editing. **Mahuan Capeletto Abdala:** Methodology, Software. **Erivelto Mercante:** Writing – review & editing, Supervision, Validation.

## Declaration Competing Interest

The authors declare that they have no known competing financial interests or personal relationships that could have appeared to influence the work reported in this paper.

## Data Availability

Data will be made available on request.
